# Euthanasia and psychiatric patients: a Spanish glance to the Dutch experience

**DOI:** 10.1192/j.eurpsy.2023.1849

**Published:** 2023-07-19

**Authors:** P. Albarracin, F. Mayor, M. Aparicio, E. Herrero

**Affiliations:** 1 Institute of Psychiatry and Mental Health, Hospital Universitario Clinico San Carlos (Madrid, Spain), Madrid, Spain

## Abstract

**Introduction:**

The recent approval of euthanasia in the Spanish legal code and its posible extension to psychiatric patients opens an unprecedented scenario in the Iberian country. We analize the experience of the Netherlands, a country where euthanasia has been in practice for over two decades, in order to foresee epidemiological trends that could be replicated in Spain.

**Objectives:**

To review the legislation on euthanasia in Spain and the Netherlands, as well as the epidemiological data regarding euthanasia applicants affected by mental health conditions in the Netherlands, to predict future epidemiological trends in a similar population in Spain.

**Methods:**

We studied the legislation on euthanasia in Spain and the Netherlands, as well as the directives of the Regional Commisions for Euthanasia in the Netherlands to analize differences and similarities between the legal codes on both countries. We also sought epidemiological data regarding the application of euthanasia on psychiatric patients in the Netherlands, gathering data from seven articles in English language obtained through a search in PubMed using the MeSH terms “Euthanasia” AND “Netherlands” and “Psychiatry”.

**Results:**

Euthanasia on psychiatric patients in the Netherlands has been a practice on the rise during the last decade, despite the elevated proportion of rejected applications and the high survival rate of this patients in later longitudinal studies. Affective disorders and personality disorders stand out as major psychiatric causes between the applicants. The Spanish legislation bears important resemblance to its Dutch predecessor, but also significant differences.

**Conclusions:**

The available data on the application of euthanasia on mental health patients in the Netherlands show an in increasing trend regarding the execution of this practice, specially on patients who gather distinct clinical features. The data provided by the Dutch experience could have some replication in Spain, as well as anticipate possible future ethical conflicts regarding the application of this service.

**Disclosure of Interest:**

None Declared

